# Supplementary figures and images for: Elevated expression of TUBA1C in breast cancer predicts poor prognosis
Source: PLoS One. 2023 Nov 30;18(11):e0263710. doi: 10.1371/journal.pone.0263710 (PMC10688681; doi:10.1371/journal.pone.0263710)

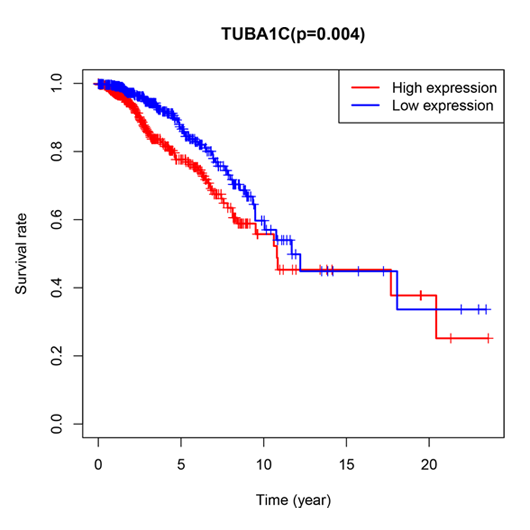

Supplement: S1 Fig — The data were processed using R software (version 4.0.3). (TIF) [file pone.0263710.s001.tif]
