# Supplementary material for: Elevated expression of TUBA1C in breast cancer predicts poor prognosis
Source: PLoS One. 2023 Nov 30;18(11):e0263710. doi: 10.1371/journal.pone.0263710 (PMC10688681; doi:10.1371/journal.pone.0263710)
Supplement: Attachment — Submitted filename: Response to Reviewers.docx [file pone.0263710.s002.docx]

Dear Dr. Suhwan Chang,

Thank you for the review of our manuscript titled: “Elevated expression of TUBA1C in breast cancer predicts poor prognosis” and for the helpful comments by the reviewer. We are pleased with the your decision in the letter dated 31/12/2021 to consider a revised version of our manuscript, addressing all the issues brought up by the reviewer.

Below, we will detail how we extensively revised the paper to address each of the reviewers' comments, numbered as they appeared in the original decision letter. Changes in the revised manuscript are highlighted in red.

Reviewer #1: This appears to be an interesting study but the foolowig changes in the manuscript are suggested.

1. There are fomatting issues, formatting should be according to PLOS guidelines. (See last line of first paragraph in the Background section).

*Response:* Thank you for the suggestion. We have modified the format of the manuscript according to the requirements stated in the PLOS ONE guidelines.

2. Cite references considering the PLOS ONE guidelines. (See second line of Discussion section).

*Response:* Thank you for the suggestion. We have modified the format of the references according to the requirements stated in the PLOS ONE guidelines.
